# Video-based instructions for surgical hand disinfection as a replacement for conventional tuition? A randomised, blind comparative study

**DOI:** 10.3205/zma001056

**Published:** 2016-08-15

**Authors:** Uwe Weber, Mihai A. Constantinescu, Ulrich Woermann, Felix Schmitz, Kai Schnabel

**Affiliations:** 1 Bern, Schweiz; 2 Inselspital Bern, Universitätsklinik für Plastische- und Handchirurgie, Bern, Schweiz; 3 Universität Bern, Institut für Medizinische Lehre Abteilung für Unterricht und Medien, Bern, Schweiz

## Abstract

**Introduction: **Various different learning methods are available for planning tuition regarding the introduction to surgical hand disinfection. These learning methods should help to organise and deal with this topic. The use of a video film is an alternative to conventional tuition due to the real presentation possibilities of practical demonstration.

**Objective: **This study examines by way of comparison which form of communication is more effective for learning and applying surgical hand disinfection for medical students in their first year of studies: video-based instruction or conventional tuition.

**Methodology:** A total of 50 first-year medical students were randomly allocated either to the “Conventional Instruction” (CI) study group or to the “Video-based Instruction” (VI) study group. The conventional instruction was carried out by an experienced nurse preceptor/nurse educator for the operating theatre who taught the preparatory measures and the actual procedure in a two-minute lesson. The second group watched a two-minute video sequence with identical content. Afterwards, both groups demonstrated practically the knowledge they had acquired at an individual practical test station. The quality (a) of the preparation and (b) of the procedure as well as (c) the quality of the results was assessed by 6 blind experts using a check list. The acceptability of the respective teaching method was also asked about using a questionnaire.

**Results: **The group performance did not differ either in the preparation (*t*=-78, *p*<0.44) or in the quality (*t*=-99, *p*<0.34). With respect to performance, it was possible to demonstrate a strong treatment effect. In the practical (*t*=-3.33, *p*<0.002, *d*=0.943) and in the total score (*t*=-2.65, *p*<0.011, *d*=0.751), the group with video-based instruction achieved a significantly better result. In response to the question as to which of the two learning methods they would prefer, the significant majority (60.4%) of students stated video instruction.

**Conclusion:** In this study, the use of the video-based instruction emerged as the more effective teaching method for learning surgical hand disinfection for medical students and is preferable to conventional instruction. The video instruction is associated with a higher learning effectiveness, efficiency and acceptability.

## Zusammenfassung

**Einleitung:** Für die Unterrichtsgestaltung zur Einführung der chirurgischen Händedesinfektion stehen unterschiedliche Lernmethoden zur Verfügung. Diese Lernmethoden sollen dabei helfen, das Unterrichtsthema zu strukturieren und zu bewältigen. Der Einsatz eines Videofilms ist, durch die realen Darstellungsmöglichkeiten der praktischen Demonstration, eine Alternative zum konventionellen Unterricht.

**Ziel: **Mit der vorliegenden Studie wird vergleichend untersucht, welche Vermittlungsform effektiver für das Erlernen und Anwenden der chirurgischen Händedesinfektion von Medizinstudenten im 1. Studienjahr ist: videobasierte Instruktion oder konventioneller Unterricht.

**Methodik: **Insgesamt wurden 50 Medizinstudierende im 1. Studienjahr per Zufall entweder der Lerngruppe „konventionelle Instruktion“ (KI) oder der Lerngruppe „videobasierte Instruktion“ (VI) zugeordnet. Die konventionelle Instruktion erfolgte durch einen erfahrenen Nurse Preceptors/Nurse Educators für den Operationssaal, der die vorzubereitenden Massnahmen sowie die eigentliche Prozedur im Rahmen einer zweiminütigen Lektion vermittelte. Die zweite Gruppe sah eine Videosequenz von 2 Minuten mit identischen Inhalten. Beide Gruppen demonstrierten ihr akkumuliertes Wissen im Anschluss praktisch an einer einzelnen praktischen Prüfungsstation. Die Güte (a) der Vorbereitung und (b) der Prozedur wurde ebenso wie (c) die Qualität des Ergebnisses von 6 geblendeten Experten anhand einer Checkliste beurteilt. Die Akzeptanz der jeweiligen Vermittlungsform wurde mittels eines Fragebogens erfragt.

**Ergebnisse: **Die Gruppenleistungen unterschieden sich weder in der Vorbereitung (*t*=-78, *p*<0.44) noch in der Qualität (*t*=-99, *p*<0.34). In Hinblick auf die Performanz konnte ein starker Treatment-Effekt nachgewiesen werden. In der Durchführung (*t*=-3.33, *p*<0.002, *d*=0.943) sowie im Total Score (*t*=-2.65, *p*<0.011, *d*=0.751) erzielte die Gruppe mit videobasierter Instruktion ein signifikant besseres Ergebnis. Auf die Frage, welche der beiden Lernmethoden sie bevorzugen würden, wurde von den Studenten mit deutlicher Mehrheit (60.4%) die Videoinstruktion angegeben.

**Schlussfolgerung: **Der Einsatz der videobasierten Instruktion stellte sich in dieser Studie als die effektivere Vermittlungsform zum Erlernen der chirurgischen Händedesinfektion für Medizinstudierende heraus und ist der konventionellen Instruktion vorzuziehen. Die Videoinstruktion ist mit höherer Lerneffektivität, Effizienz und Akzeptanz assoziiert.

## 1. Introduction

Various different learning methods are available for planning tuition regarding the introduction to surgical hand disinfection. These learning methods should help to organise and deal with this topic. A learning method should be selected that is suitable for teaching the topic. The transfer of nosocomial infectious agents takes place most frequently via the hands of staff. The prevalence studies for nosocomial infections revealed that an extrapolated 70,000 patients in Switzerland suffer from nosocomial infection, of whom 2,000 die [1]. The risk in the event of a perforation of surgical gloves can be reduced by correct surgical hand disinfection [2]. In her paper, Cloyd describes how students are inadequately prepared for work in the operating theatre and how they have the feeling that they are a burden and wish to have more effective preparation [3]. The question arises here as to how preparation for work in the operating theatre can be improved and which instruction options are available. In her paper “Basic Aseptic Techniques (BAT)”, Leeper describes the entrance level for the operating theatre. Maintenance of the sterile OP environment and surgical hand disinfection were named here as the two most important points for the entrance level that medical students must demonstrate. She describes how a reduction in post-operative wound infection was achieved as the outcome as a result of knowledge of BAT training [4]. 

The following learning methods are available for the introduction to surgical hand disinfection:

1. Classical theoretical teaching with aims and objectives without any possibility of practical demonstration under guidance. This tuition can be supported by literature and image material.
2. Self-study of the literature and image material without a practical demonstration.
3. Theoretical input on the topic with a demonstration of surgical hand disinfection by a trained specialist (nurse preceptor/nurse educator), with subsequent opportunity for the students to practise in a simulated or real surgical unit. This method with a trained specialist (nurse preceptor/nurse educator) and subsequent opportunity to practise was also named the most often as the first choice for tuition for the skills (Leeper).
4. The use of a video film, an alternative to the real presentation possibilities of practical demonstration. By using a video film, facts and connections can be illustrated better visually with the aid of digital text, graphics and animation and shown in a shorter time.

People are visually inclined and visual intelligence plays a most important role [5]. The use of videos has many other advantages, not investigated in this study [6], such as the option of repeated viewing [7] and in specific preparation for examinations [8]. The use of video instructions has been shown in literature to have equally good or mostly positive effects in the teaching of skills in all fields. As early as 1993, Yoder showed that the video group demonstrated greater learning success when learning the cognitive principles of aseptic methods [9]. In a comparison between didactics, video instruction and computer-based training, the video instruction demonstrated a significant improvement in technical skills [10]. Xeroulis et al. demonstrated that video instruction with expert feedback can be an effective method for instructing basic technical skills [11]. In a comparison between computer-based video instruction and conventional tuition for learning basic surgical skills of suturing and knotting the medical students demonstrated no differences when learning the method [12]. In a further study by Shippey, three different learning methods, video instruction, practical with an instructor and independent learning, for learning suturing techniques were compared to each other. The results demonstrated significantly better effects in the case of video instruction [13]. The use of video instruction for simulation with a venous port catheter demonstrated that cognitive and technical knowledge increased [14]. 

### 1.1. Question

- To what extent can video instruction for surgical hand disinfection be just as effective as conventional instruction?

In this non-inferiority study we investigated which method a (VI=video instruction) or b (CI=conventional instruction) had the better effect for learning surgical hand disinfection among first-year medical students at the University of Bern. Based on the literature mentioned in the introduction, we expect that the students will demonstrate at least an equivalent or better performance after the video instruction (VI) than the students from the conventional instruction (CI). 

The directional hypothesis is thus: H(1):VI≥KI. The null hypothesis is: H(0):VI<KI

## 2. Methodology

### 2.1. Random Sampling and Design 

All 208 human medical students in the first semester at the University of Bern were sent a letter in the autumn of 2011 inviting them to take part in a voluntary study to examine two different teaching methods. The participants were not told the exact content of the teaching methods. 50 human medicine students signed up: 27 men and 23 women. The students were randomised [http://www.psychicscience.org] and divided into the observation groups Video Instruction (VI) and Conventional Instruction (CI). 

**Design: **Two different groups of students were compared (between-subjects). The verification of the learning results by the experts was blind.

**Description of treatment: **an educational film regarding conduct in the operating theatre was developed to investigate the question [http://e-learning.studmed.unibe.ch/chirosurf/htmls/slide.html?chirosurf%7Coperation%7Ctheater%7Corganisation%7C1]. This educational film contains a commentated section that is nearly two minutes long (minute: 6:35-8:25) with visual overlays about surgical hand disinfection, which was used for investigating the question. 

An experienced nurse educator for the operating theatre was provided with the same text and content as in the film. The nurse educator was tasked with teaching the same content in a two-minute instruction with simultaneous demonstration of surgical hand disinfection. 

#### 2.2. Instruments

A check list for assessing surgical hand disinfection for recording the success of the teaching surrounding surgical hand disinfection was modified according to a template by Wilkinson [15] and adapted to the latest handling methods [16] (c.f. Attachment 1: Check List for Surgical Hand Disinfection ). The checklist was submitted to three experts (one hygienist and two surgeons) who saw no need for further adjustments following these adaptations. It was possible to achieve the maximum scores shown in Table 1 (Tab. 1).

Six experts worked through the items on the check list on tablets (iPad ®) using the OSCE-Eval [http://www.e-osce.ch/] application. All six advisors were blind with respect to the students’ instruction method (conventional or video-based) and were instructed beforehand regarding the check list with the observational criteria and the assessment. The students’ level of knowledge regarding surgical hand disinfection was evaluated using an additional questionnaire. After the investigation, the test persons were asked how satisfied they were with the different learning methods and which learning method (video, tutorial or both) they would prefer.

#### 2.3. Practice

##### 2.3.1. Intervention 

The interventions were started at the same time in two different rooms. The VI group (video instruction) was shown the two-minute video sequence as an introduction to surgical hand disinfection. The CI group (conventional instruction) was taught using the conventional lesson as has been customary to date with the same contents concerning the introduction to surgical hand disinfection by the nurse educator with a simultaneous demonstration. Carrying out the conventional instruction was likewise limited to two minutes. Afterwards the students from both groups were called up according to the allocation scheduled beforehand by the random generator to carry out the practical demonstration at an individual practical test state at the **B**ern **i**nterdisciplinary **S**kills and **S**imulated Patients Centre (**BiSS**) at the Institute for Medical Studies at the University of Bern (IML). Six test stations set up identically with the required materials (with hand disinfectant mixed with Visirub©, mask, protective glasses, UV lamp) were provided. Five minutes were calculated for each run (see Table 2 (Tab. 2)). One minute for orientation and preparation with the required material that the students had to select themselves. Three minutes to correctly carry out the surgical hand disinfection in good time. One minute to check the quality using the UV lamp. During the demonstration the preparation, practical and quality, as described, were assessed using the check list that had been compiled. Following the demonstration, the students attended the instruction of the respective other group. There they were presented with the respective other teaching method within the same timeframe. After the presentation, the students were asked about the different learning methods using our questionnaire (see Attachment 2: Questionnaire ) and about their satisfaction and acceptability. 

## 3. Results

The results of the 50 students were analysed. The statistical analysis of the data was carried out with the aid of Microsoft Office Excel 2010 and SPSS 20 for Windows.

If one compares the participants in the video instruction (VI) with the conventional instruction (CI) in detail (Table 3), the following picture emerges in the observation areas: in the preparation, the participants from VI group did not perform any differently to the CI group. In the practical, the VI group performed significantly better than the CI group with an effect size of 0.943 (Cohen’s d). In quality, the VI group did not perform any differently to the CI group. In the total score, a significantly better result is demonstrated by VI group with an effect size of 0.751 (Cohen’s d) (see Table 3 (Tab. 3)). 

In the T-test (see Table 4 (Tab. 4)) a significant influence of the video instruction on the practical is demonstrated (*p*=0.002) and in the total score (*p*=0.011). 

### 3.1. Interpretation of the Questionnaire

The interpretation of the questionnaire on satisfaction with and the acceptability of the learning methods was voluntary and anonymised. We received 48 completed responses from the 50 participants. In the interpretation of the question regarding prior knowledge, it was ascertained that 7 out of 23 students from the CI group had already been taught surgical hand disinfection. In the VI group it was 3 out of 25 students who likewise made this statement (see Table 5 (Tab. 5)).

In response to the question as to which of the interventions they would prefer, 29 (60.4%) answered in favour of the video and 19 (39.6%) in favour of the lesson. Therefore, five students from the conventional instruction group decided on the video having watched the video (see Table 6 (Tab. 6)).

In response to the question as to which methods: lesson, video or both, they regarded to be suitable for the preparation for surgical hand disinfection “both” was clearly selected with 37 responses (see Table 7 (Tab. 7)).

## 4. Discussion

The use of video instruction demonstrates, as described in the literature referred to, a positive effect in skills acquisition. Cognitive and technical skills can be improved using the real presentation possibilities of practical demonstration. 24 students were successfully taught in two minutes using the video instruction. If one regards the total expenditure in comparison and the possibility of repeated use, efficiency speaks clearly in favour of video instruction as a result of the greater effectiveness. When interpreting the questionnaire with regard to the question as to which learning method: video instruction or conventional instruction, they would prefer, it emerged that 17 students from the CI group decided in favour of the video and 8 in favour of the lesson. It was surprising that 2/3 of the CI group would prefer video instruction. All of the students selected the decision regarding the other learning method without knowing the results of their demonstration at the practical test station. In the VI group the result was balanced; 12 students preferred video instruction and 13 students preferred conventional instruction. Overall, it was expected that the respective learning method experienced before the practical test would be preferred by the majority. That the video instruction was preferred by the majority is in accord with the results in the literature cited in the introduction and can be ascribed to the known advantages of video instruction (general preference for digital media among the younger generation, the highlighting of key elements and standardisation of procedures) [6]. A clear statement in the interpretation was that a combination of conventional instruction and video instruction was regarded by the students as the ideal teaching unit. This was not unexpected, since an addition of both learning methods is naturally regarded to be a potential advantage by students. Some questions raised during the practical should be considered in further studies: would the differences in the practical between the two intervention groups possibly turn out differently if one had used several tutors each with a smaller group instead of one tutor? Would other learning methods, for example a demonstration with subsequent opportunities to practice in a simulated or real operating theatre, literature with the use of image material, lead to different results? It should also be considered critically that it was more the skills that were being tested in the practical test station and the students in both groups were only taught once for two minutes with the option of assisted practicals, by means of personal feedback and without any chance to ask questions. 

## 5. Conclusions

We can answer our question as to whether video instruction for surgical hand disinfection can be just as effective as conventional instruction in the affirmative and reject the null hypothesis. In the evaluation going beyond the pure question, a positive result is demonstrated in all areas in favour of video instruction. In the preparation and in the quality both groups achieved equally good results. The video instruction demonstrated a significantly better effect in the practical and in the total score. The evaluated sequence of surgical hand disinfection in the educational film demonstrates an effective and efficient transfer of knowledge. In response to the question as to which of the two methods they would prefer, the video instruction was selected clearly. A further relevant aspect is that a video film represents a meaningful enhancement to the choice of learning as a result of more extensive possibilities of presenting the teaching content and can increase cognitive and technical knowledge. Video instruction provides the opportunity to present relevant learning content realistically and for use in independent studies. Videos help the students to repeat learning content from the lesson independently at their own speed. Looking to the future, the study of the long-term effect and the different teaching methods would be of interest.

## 6. Acknowledgements

I would like to thank the following people most sincerely for their support during this project:

- Mr Giovanni Ferrieri, Multimedia Designer Video
- Mr Jürgen Schmidt, Dipl.-Pflegw. (Technical University)
- Ms Margrit Catani and Ms Diana Schuler
- Dr. phil. nat. Banu Yürüker, MME
- Ms Sabine Richter, Technical Assistant
- Ms Gudrun Stopper, MME
- Mr Stephan Schallenberger, MAS in HCID
- Mr Kevin Gaunt, iOS Software Developer and Research
- Barbara Haldemann, Head of Gynaecological Operating Theatre at the Inselspital Bern

## Competing interests

The authors declare that they have no competing interests.

## Supplementary Material

Check list for surgical hand disinfection

Questionnaire

## Figures and Tables

**Table 1:**
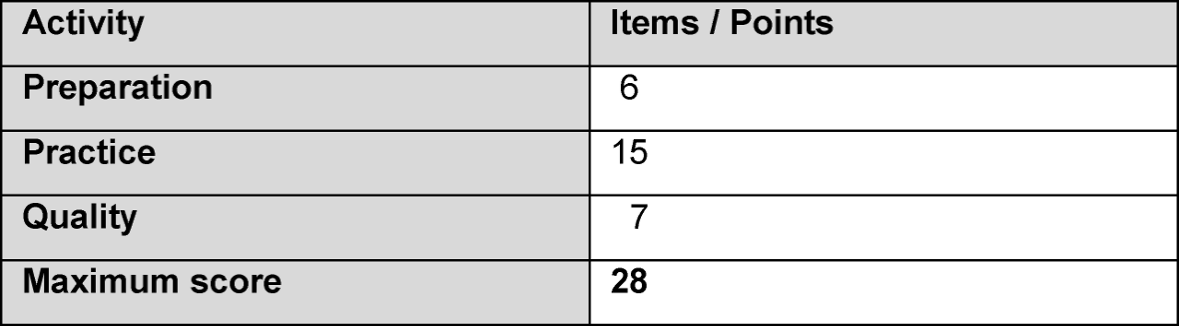
Distribution of points

**Table 2:**
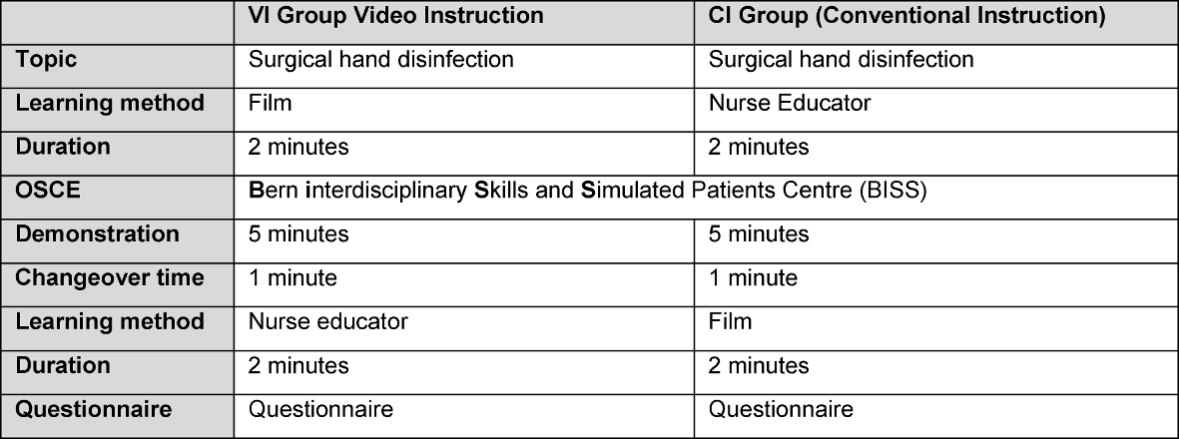
Process Schedule

**Table 3:**
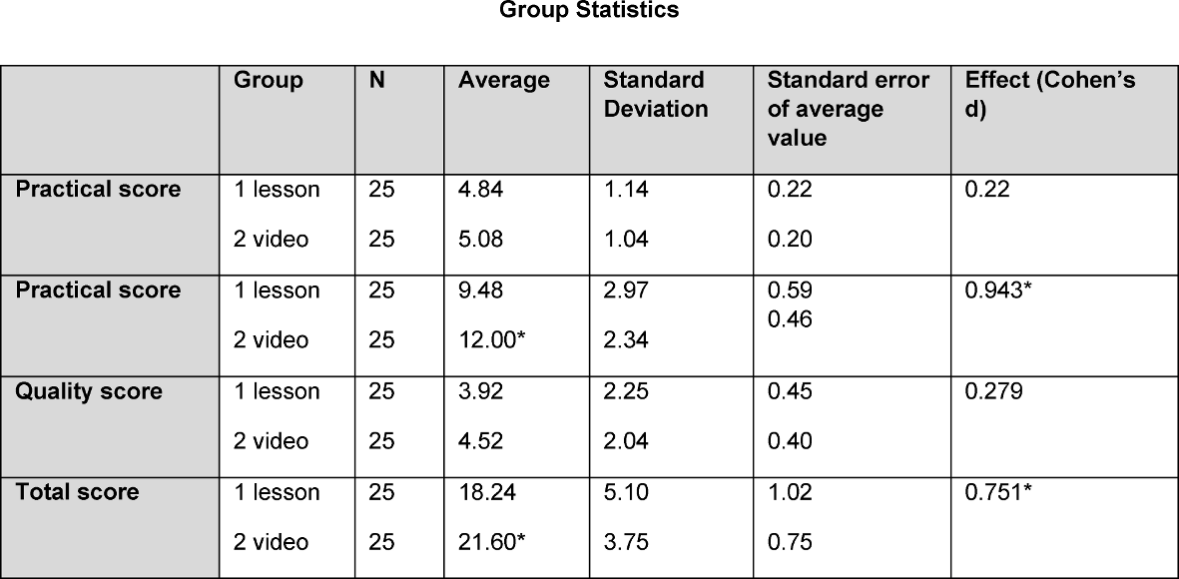
Descriptive statistics of the observation dimensions separated by intervention group

**Table 4:**
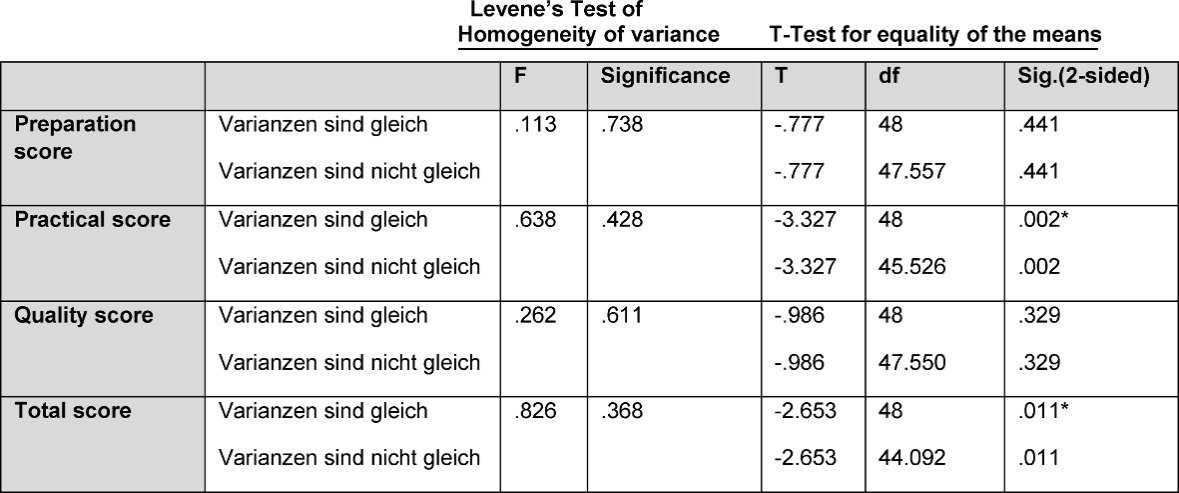
Results of the T-test

**Table 5:**
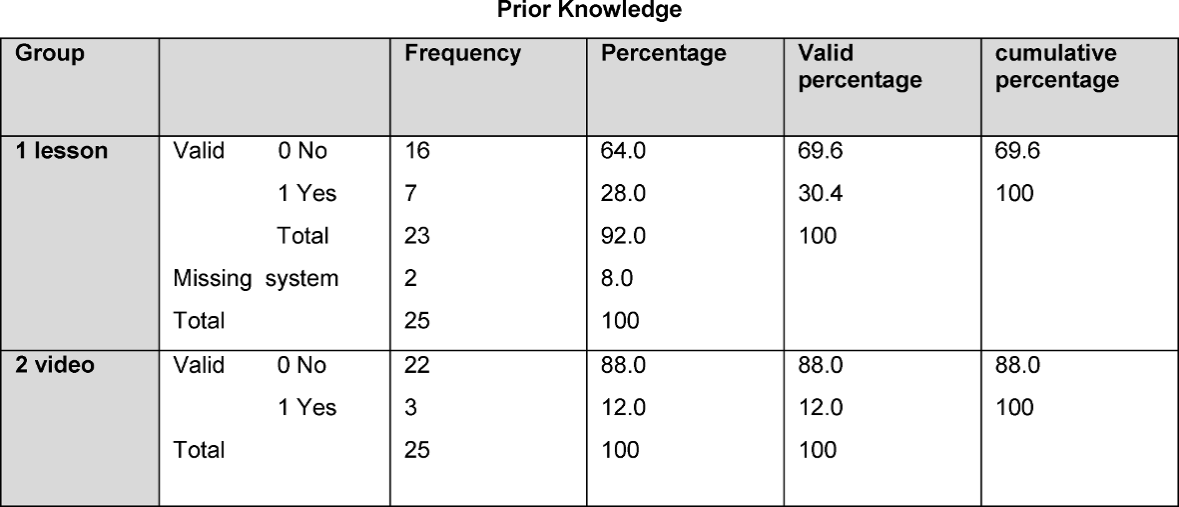
Results of prior knowledge

**Table 6:**
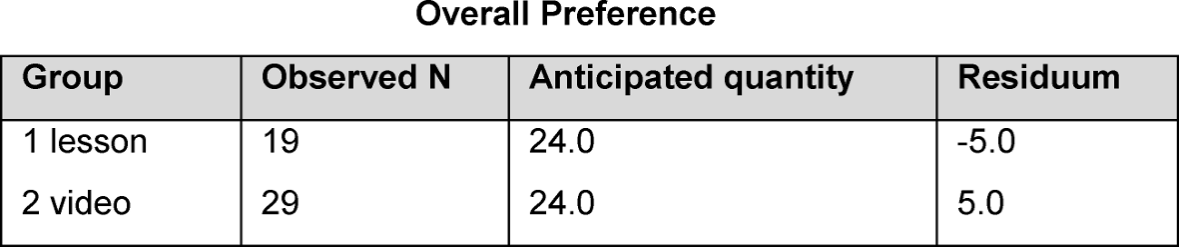
Overall preference

**Table 7:**
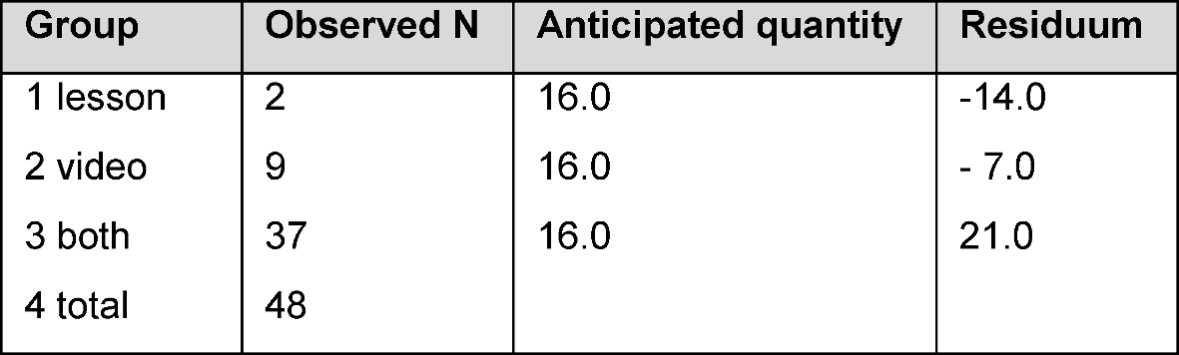
Suitability of learning method for content
