# Supplementary material for: Video-based instructions for surgical hand disinfection as a replacement for conventional tuition? A randomised, blind comparative study
Source: GMS J Med Educ. 2016 Aug 15;33(4):Doc57. doi: 10.3205/zma001056 (PMC5003145; doi:10.3205/zma001056)
Supplement: Check list for surgical hand disinfection [file JME-33-57-s-001.pdf]

Surgical hand disinfection with a disinfectant containing alcohol		
**Assessment**	**No**	**Yes**
	0	1
**Preparation:**		6 points
The hands have no wounds, nails are cut short, are clean and without nail varnish (**female**)		
No rings or watches		
Cap fitted correctly		
Mask fitted correctly		
Eye protection has been put on		
**No** pre-cleaning of the hands with soap and water		
**Surgical hand disinfection**		15 points
Disinfectant is taken out with elbow		
**1st minute, from finger tips up to the elbows**		
**2nd minute, from the finger tips to the middle of the lower arm**		
**3rd minute, only in the area of the hands themselves disinfected.**		
Fingers		
Between the fingers		
Palms of the hands		
Thumbs		
Total application time and rules are complied with		
**Correct position of hands is complied with:** Hands and arms bent at the elbows		
**Correct position of hands is complied with:** Hands are held vertically upwards		
**Correct position of hands is complied with:** The hands are in the highest position		
**Sufficient disinfection is used,** arms and hands are always moist		
**Sterility is ensured** no touching of unsterile area		
Letting disinfectant take effect and dry and not wiping it off.		

**Assessment**	**No**	**Yes**
	**1**	**0**
**Quality of disinfection**<br>**Residue on:**	**7 points**	
Fingertips		
Between fingers		
Palms of hands		
Between fingers and thumbs		
