# Supplementary material for: Video-based instructions for surgical hand disinfection as a replacement for conventional tuition? A randomised, blind comparative study
Source: GMS J Med Educ. 2016 Aug 15;33(4):Doc57. doi: 10.3205/zma001056 (PMC5003145; doi:10.3205/zma001056)
Supplement: Questionnaire [file JME-33-57-s-002.pdf]

Questionnaire

The questionnaire for personal feedback at the end of the two learning methods contained the following questions:

1. Your gender
   ☐ Woman ☐ Man

2. Had you already been taught surgical hand disinfection before this study?
   ☐ Yes ☐ No

3. Which learning method would you regard to be ideal for the introduction to surgical h and disinfection?
   ☐ Lesson
   ☐ Video
   ☐ Both

4. If you could only choose one learning method, which learning method would you have attended?
   ☐ Lesson ☐ Video
